# Rule-Based Model of Vein Graft Remodeling

**DOI:** 10.1371/journal.pone.0057822

**Published:** 2013-03-22

**Authors:** Minki Hwang, Marc Garbey, Scott A. Berceli, Rongling Wu, Zhihua Jiang, Roger Tran-Son-Tay

**Affiliations:** 1 Departments of Mechanical & Aerospace Engineering, University of Florida, Gainesville, Florida, United States of America; 2 Department of Computer Science, University of Houston, Houston, Texas, United States of America; 3 Department of Surgery, University of Florida College of Medicine, Gainesville, Florida, United States of America; 4 Malcom Randall Veterans Affairs Medical Center, Gainesville, Florida, United States of America; 5 Center for Statistical Genetics, Division of Biostatistics, Pennsylvania State University, Hershey, Pennsylvania, United States of America; The Ohio State University, United States of America

## Abstract

When vein segments are implanted into the arterial system for use in arterial bypass grafting, adaptation to the higher pressure and flow of the arterial system is accomplished thorough wall thickening and expansion. These early remodeling events have been found to be closely coupled to the local hemodynamic forces, such as shear stress and wall tension, and are believed to be the foundation for later vein graft failure. To further our mechanistic understanding of the cellular and extracellular interactions that lead to global changes in tissue architecture, a rule-based modeling method is developed through the application of basic rules of behaviors for these molecular and cellular activities. In the current method, smooth muscle cell (SMC), extracellular matrix (ECM), and monocytes are selected as the three components that occupy the elements of a grid system that comprise the developing vein graft intima. The probabilities of the cellular behaviors are developed based on data extracted from in vivo experiments. At each time step, the various probabilities are computed and applied to the SMC and ECM elements to determine their next physical state and behavior. One- and two-dimensional models are developed to test and validate the computational approach. The importance of monocyte infiltration, and the associated effect in augmenting extracellular matrix deposition, was evaluated and found to be an important component in model development. Final model validation is performed using an independent set of experiments, where model predictions of intimal growth are evaluated against experimental data obtained from the complex geometry and shear stress patterns offered by a mid-graft focal stenosis, where simulation results show good agreements with the experimental data.

## Introduction

Vein bypass grafting is one of the primary treatment options for arterial occlusive disease. Although it provides satisfactory results at an early stage of treatment, intermediate to long-term failures are common and patency can be limited to a few months [1]–[3]. When vein segments are implanted into the arterial system, they adapt to the higher blood flow and pressure through thickening and expansion of the wall [4]–[6]. Although this early response is not considered pathologic, it is believed to be the foundation for later vein graft failure. The acute alteration in biomechanical forces, namely wall shear stress and intramural wall tension, have been identified as the dominant factors that initiate and propagate the cascade of intersecting biologic events that dictate the ultimate configuration of the graft [7].

Following harvest of the vein segment, ex vivo manipulation, and re-implantation in the arterial circulation, a well-defined sequence of repair and remodeling events are initiated. Early injury to the medial smooth muscle cells (SMC) leads to a burst in apoptotic cell death that peaks at three days and is resolved by one week [8]. Starting at one week and continuing through one month, repair is initiated by the influx of macrophages and the migration and proliferation of SMC [9]. Co-incident with these events is the local degradation of the extracellular matrix (ECM), which facilitates the detachment and mobilization of the SMC to the developing intima. In the weeks to months following implantation, continued expansion of the intima is accomplished by the conversion of SMC to a synthetic phenotype and the robust synthesis and deposition of extracellular matrix into the wall [10]. Modulating this cascade of events are the local biomechanical forces that regulate both gene expression and receptor-matrix interactions in the wall [11]–[14].

Modeling of dynamic systems has been classically accomplished using a series of differential equations, which dictate the relative time-dependent changes in the key elements within the system. While such mathematical models are relatively simple and provide explicit relationships among the variables, they often fail to yield much insight into the complex interactions that are inherent in most dynamic systems. Recent work from our group has defined the temporal changes in wall thickening and outward expansion for implanted vein grafts and mapped their relationships through the range of physiologic wall shear and tensile forces [15], [16]. While useful in understanding the dynamic interplay between shear and wall tension as driving forces for remodeling, this analysis fails to provide a mechanistic understanding that is necessary for targeted therapeutic intervention [17].

Rule-based modeling approaches, such as agent-based modeling, utilize the fundamental understanding of individual elements to predict emergent behavior within complex systems [18]–[20]. Through the integration of targeted experimental data and insight that has been accumulated in the literature, a simple set of rules are developed and applied to the computational domain to simulate and predict system dynamics. In the current manuscript, a rule-based simulation of vein graft adaptation, and the impact of the local hemodynamics on graft remodeling, is developed. Serving as the basic building blocks for vein graft repair, SMC and ECM populate a rectangular grid system, where the influence of SMC replication, SMC apoptosis, and ECM deposition on regional graft morphometry is tracked. Using kinetic data obtained from rabbit vein grafts that are exposed to a range of hemodynamic conditions, the probabilities of various behaviors are applied to SMC and ECM to determine the next state for each of these elements. Following initial feasibility testing employing a 1-dimensional (1-D) domain, methodology and results for a 2-dimensional (2-D) algorithm are provided. Lastly, validation of the model in a complex, realistic geometry is provided through correlation of model predictions against histomorphometric measurements of harvested vein grafts.

## Methods

### Animal Experiments of Vein Graft Remodeling

Over the last decade, our laboratory has developed and validated a unique vein graft construct in which rabbit carotid vein grafts are exposed to a range of divergent hemodynamic conditions [5],[10],[21]–[23]. Briefly, New Zealand White rabbits (3.0–3.5 kg) underwent bilateral jugular vein interposition grafting and unilateral distal carotid artery branch ligation to create two distinct flow regimes, which result in an immediate 10-fold difference in blood flow rates between the grafts. Following implantation times ranging from 1 to 28 days [1 day (n = 8), 3 days (n = 7), 7 days (n = 8), 14 days (n = 5), 28 days (n = 6)], grafts were re-explored, flow rate and intraluminal pressure measured, and tissue harvested for histomorphologic measurements. Excised grafts were fixed in 10% formalin, paraffin embedded, and histologically sectioned for further analysis.

To facilitate identification of those cells undergo active proliferation, rabbits were provided a subcutaneous injection of bromodeoxyuridine (BrdU; 50 mg/kg) twenty-four hours prior to graft harvest. Histologic sections were assayed using an antibody specific for BrdU (BrdU Staining Kit, Zymed, San Francisco, CA) and quantitative analysis of microscopic images (40x) performed to identify the fraction and distribution of SMC undergoing cell division. Morphologic measurements on Masson stained histologic specimens were completed to determine intimal thickness and lumen diameter, as previously described [21]. Wall shear stress (τ) in each graft was estimated via Poiseuille's Law (τ = 4 µQ/πR^3^), where μ = viscosity (0.035 poise), Q = flow rate, and R = lumen radius.

To evaluate the accuracy of the agent-based model when challenged with the local flow disturbances associated with a complex geometry, a rabbit vein graft construct with a high grade focal stenosis was utilized. Through use of a 3F (0.95 mm external diameter), externally placed, polyethylene mandrel and an 8-0 silk suture ligature in the mid-portion of each graft, an 80% focal reduction in lumen cross-sectional area was created. Grafts were harvested at 28 days, perfusion-fixed with 10% formalin, embedded in paraffin, and histologically sectioned at 5 µm intervals. Every tenth section was collected, with the location of each section recorded with reference to the stenotic ligature. Sections were stained using Masson's trichrome, and morphologic measurements obtained.

All studies were approved by a local Institutional Animal Care and Use Committee and conformed to the Guide for the Care and Use of Laboratory Animals (National Research Council, 2011).

### Probability of Smooth Muscle Cell Division

**Figure 1:**
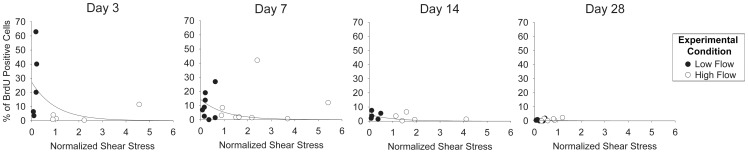
Percentage of BrdU Positive Cells as a Function of Shear Stress and Time. Shear stress is normalized with respect to the pre-ligation shear, which was measured to be 10 dynes/cm^2^. BrdU was injected one day prior to harvest. Filled and open circles are the data from the low and high flow side of the bilateral vein graft experiment, respectively.

The influence of wall shear stress and implantation time on SMC proliferation in the developing intima is provided in Figure 1. Regression analysis demonstrated the fraction of BrdU positive nuclei in the intima to be exponentially related to both shear stress and implantation time, yielding the following expression [24], [25]:
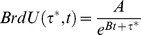
(1)where *BrdU* is the percentage of BrdU positive SMC nuclei, *t* is time (days), and τ* is wall shear stress (normalized to the average pre-ligation shear stress of 10 dynes/cm^2^). Least squares regression analysis against the experimental data provided values of 44% and 0.16 day^−1^ for coefficients *A* and *B*, respectively. General agreement between the model estimation and the experimental data is observed (R^2^ = 0.22).

Due to the limited duration of S-phase during which BrdU is incorporated into the DNA and the change in cell number that occurs during the 24 hour interval from BrdU injection to graft harvest, the probability of cell division cannot be assumed to be the simple percentage of stained nuclei. Assuming: 1) BrdU is metabolically available to the vasculature for two hours following injection [26], 2) the cell cycle time is 24 hours, 3) BrdU integration into genome occurs only during S-phase, and 4) G1, S, and G2/M phases each takes up one-third of the cell cycle time (see Supplementary Information S1) [13], the space-averaged probability of cell division (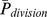) during a 1-hour period can be written:
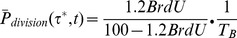
(2)where T_B_ is the time between BrdU injection and tissue harvest, which is 24 hours in the present study.

The fraction of BrdU positive cells varies throughout the vein graft, with the highest rate of cell division within the most superficial portions of the developing intima. The relationship between SMC proliferation and distance from the luminal surface was extrapolated using previous data from our laboratory [8]. The following form for the spatial distribution of cell proliferation, 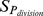, was extracted from the experimental data using the following expression
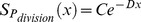
(3)where *x* is distance from the luminal surface and *C* and *D* are constants.

Combining the expressions for the space-averaged probability of cell division (Eq. 2) with the spatial distribution of cell proliferation (Eq. 3) yields the probability of cell division (*P_division_*) as a function of wall shear, time after implantation, and distance from the lumen
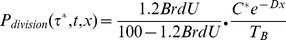
(4)Coefficient *D*, which is independent of shear, time, or distance, was obtained through regression of Eq. (3) to the experimental data and is equal to 27.3 mm^−1^. C^*^ was calculated at each time interval and is acquired by integrating Eq. (4) over the intimal thickness (*IT*) and equating it to the space-averaged proliferation probability multiplied by the intimal thickness at each time, as shown below
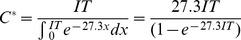
(5)


A more detailed derivation of these equations for cell division probability is provided in the Supplementary Information S1.

### Probability of Smooth Muscle Cell Apoptosis

The terminal deoxynucleotidyltransferased UTP nick end labeling (TUNEL) assay offers the most reliable *in situ* assessment of cell apoptosis and was used in the current study. Through the integration of literature based experimental values [14], the probability of SMC apoptosis was estimated. Similar to the cell proliferation analysis, an expression for TUNEL positivity was obtained using the following exponential form:
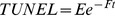
(6)where regression against experimental data yields value of 5.0% and 0.32 day^−1^ for coefficients *E* and F, respectively.

Assuming the duration of apoptosis to be comparable to the cell cycle time, the same correlation used in the cell division probability analysis was used for calculation of the space-averaged probability of cell apoptosis (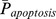) and can be written as follows:
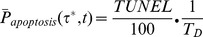
(7)where *TUNEL* is the percentage of TUNEL positive cells and *T_D_* is the amount of time the dead cells remain in the tissue before being removed, which is assumed to be 24 hours in the current analysis [27]. Unlike SMC proliferation, where replicating cells are predominately located adjacent to the lumen, SMC apoptosis occurs in the outermost portions of the graft intima. To determine the spatial distribution of apoptosis (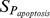), an approach analogous to that used for the cell proliferation analysis was applied except for the substitution of 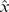 (the distance from the outside of the intima) for *x* (the distance from the lumen), to yield the following expression:
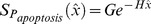
(8)where *G* and *H* are constants.

Combining the expressions for spatial distribution of SMC apoptosis (Eq. 8) with the space-averaged probability of apoptosis (Eq. 7), yields the probability of cell apoptosis (*P_apoptosis_*) as a function of wall shear, time after implantation, and distance from the outside of the intima wall as follows:
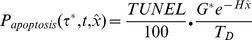
(9)Coefficient *H* was obtained through regression of Eq. (8) to the experimental data and was equal to 6.7 mm^−1^. *G* ^*^ at each time interval was acquired by integrating Eq. (9) over the intimal thickness (*IT*) and equating it to the space-averaged apoptosis probability multiplied by the intimal thickness at each time, to yield the following expression
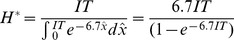
(10)A more detailed derivation of these equations for cell apoptosis probability is provided in the Supplementary Information S1.

### Extracellular Matrix Production and Degeneration

The volume percentage of SMC in vein graft intima ranges from 20–40% and remains constant during early graft adaptation [6],[7],[28]. Based on experimental results from our laboratory, the following assumptions are made:

- The initial vein at implantation is composed of 25% SMC and 75% ECM, organized in a random distribution in the wall;
- Up to four ECM elements are produced following each SMC division. When an SMC divides, each of the two daughter cells can produce one matrix element 24 hours after cell division and a second matrix element 24 hours later if it does not undergo cell division within that interval.
- When a cell undergoes apoptosis, four matrix elements nearest to the cell are removed from the computational domain.

Initial testing of this schema through 28 days using the one-dimensional simulation (see below) demonstrated a fractional SMC volume of 0.27. Also resulting from the integration of these matrix-based rules with the SMC division expressions (Eq. 4 and 5) was a variable density of SMC across the wall. With the higher probability of a SMC element undergoing a second cell division event before producing a total of four matrix elements, an increased density of SMC near the lumen is predicted, a finding consistent with experimental observations [8].

### Monocyte Influx and Augmented Matrix Production

Monocyte influx into the wall has been identified as a critical regulator of the vein graft adaptive response. Based on experimental work in our laboratory [29], there is a shear-dependent, early burst in monocyte influx that rapidly tapers to baseline. Founded in this observation, the same model form to describe the time and shear dependence of cell proliferation (Eq. 2) is used to predict the net rate of monocyte entry into the vein graft wall.
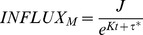
(11)where *INFLUX_M_* is the net rate of monocyte entry, and *J* and *K* are constants. The exponential dependence on time is assumed to be similar to SMC replication, so that *K* is assigned the same value (0.16 day^−1^) as that used previously (Eq. 1). The monocyte content (normalized to total cell number) approximates 5% in vein grafts during exposure to normal shear conditions, yielding a value for *J* of 175/(mm^2^·hour) at t = 0. At time zero, monocytes are randomly distributed throughout the initial intima. Monocytes entering the intima after the start of the simulation are placed at random positions within the grid.

Following vein graft implantation, Hoch et al. [30] showed that macrophage depletion suppresses thickening of the wall with no significant change in the total number of SMC. This and other evidence suggests that monocytes provide an importance source for growth factors and cytokines, acting to augment SMC matrix production with limited influence on the rate of SMC proliferation. As a first approximation, the effects of macrophages were incorporated into the model through augmentation of matrix synthesis at the time of SMC division. Specifically, each daughter cell creates two ECM elements that are placed randomly in one of the four grid positions adjacent to the dividing cell according to the following expression:
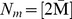
(12)where *N_m_* is the number of matrix elements generated by each of the 2 new daughter cells, 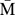 is the normalized monocyte entry rate (i.e. a given monocyte entry rate (M) divided by the monocyte entry rate at normal condition (M_0_) from Eq. 11), and [ ] denotes rounding to the nearest integer.

A summary table of the experimental datasets, secondary variables, and coefficients used in development of the various model components are provided in Tables 1, 2, and 3, respectively. Table 4 provides a summary overview of the assumptions used in the current model.

**Table 1:** Experimental datasets used in model development.

Experimental Parameter	Description	Functional Dependence
*BrdU*	Percentage of SMC nuclei incorporating BrdU [8]	*f* (τ*, t, x)
*TUNEL*	Percentage of SMC undergoing apoptotic cell death [14]	*f* (τ*, t, 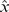 )
*IT*	Intimal thickness (µm) [5], [21]	f (τ*, t)

**Table 2:** Secondary variables used in model development.

Variable	Description
τ	Wall shear stress
*Q*	Flow rate
*R*	Lumen radius
*t*	time
τ*	Wall shear stress (normalized to the pre-implantation average shear stress of 10 dynes/cm^2^)
*x*	Distance from the outside of the intima
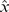	Distance from the luminal surface of the graft
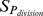	spatial distribution of SMC division
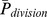	space-averaged probability of SMC division
*P_division_*	probability of SMC division apoptosis
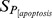	spatial distribution of apoptosis
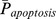	space-averaged probability of SMC apoptosis
*P_apoptosis_*	probability of cell apoptosis
*INFLUX_M_*	net rate of monocyte entry into the intima

**Table 3:** Model coefficients derived through regression analysis of primary experimental data.

Coefficient	Description	Value
*A*	BrdU labeling coefficient at t = 0	44%
*B*	BrdU time decay coefficient	0.16/day
*C*	BrdU labeling coefficient at x = 0	23.8%
*D*	BrdU spatial-decay coefficient	27.3 mm^−1^
*E*	TUNEL labeling coefficient at t = 0	5.0%
*F*	TUNEL time decay coefficient	0.32/day
*G*	TUNEL labeling coefficient at x = 0	19.3%
*H*	TUNEL spatial-decay coefficient	6.7 mm^−1^
*J*	Maximal monocyte entry rate at t = 0	175/(mm^2^•hour)
*K*	Monocyte entry rate decay coefficient	0.16/day

**Table 4:** Model assumptions.

Category	Assumptions
BrdU	BrdU is available for the first 2 hours after injection [26].
Cell Cycle	Cell cycle time is 24 hours, and G1, S, and G2/M phases each takes up one-third of the cell cycle time [13].
Cell Size and Ratio	Cell size is 7 µm×7 µm. Initial volume ratio of cell to matrix is 1∶3.
Apoptosis	Duration of apoptosis is 24 hours [27].
Matrix Production	At the time of cell division, each of the two new daughter cell elements produces one matrix element 24 hours after division, and another one 24 hrs later if it does not divide itself during that period.
Macrophage Effect	At the time of cell division, each of the two new daughter cell elements produces an additional matrix element.

### Agent-Based Modeling: One-Dimensional Algorithm

For initial model feasibility testing and parameter validation, a one-dimensional Cartesian grid system was employed. This 1-D construct consisted of a single column of elements where each element represented either a SMC or an ECM. Elements in the grid were uniform in size (7 µm), to approximate the dimensions of a SMC. Consistent with the baseline thickness of the non-diseased venous intima, the initial configuration consisted of a single SMC element. The simulation was initiated using the following reorganization rules:

- When an element is created, via SMC replication or ECM production, it is assigned to one of the two adjacent spaces with equal probability.
- To provide space for new element in the grid, existing elements are shifted towards the lumen.
- When an element is lost, via SMC apoptosis or ECM degradation, the resulting void is occupied through movement of the remaining elements in a downward (outside) direction.

### Agent-Based Modeling: Two-Dimensional Algorithm

**Figure 2:**
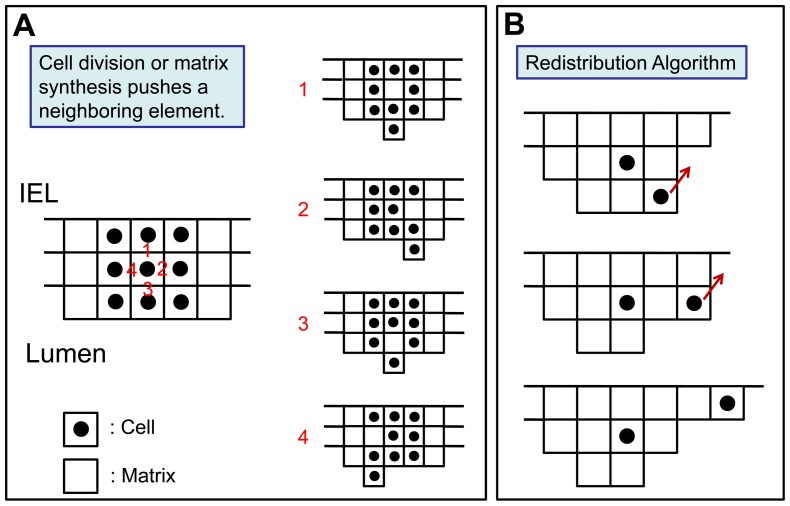
Schematic Demonstrating Element Movement and Redistribution in a 2-D Domain. A. When a smooth muscle cell undergoes division, four new matrix elements are inserted into the grid. The new elements can be produced from each of the 4 sides of the cell. In each of the four scenarios when a matrix element is produced, the new matrix element pushes the adjacent cells in the direction of lumen. B. Element redistribution algorithm, demonstrating the shifts which occur when neighboring columns manifest a greater than 2-element difference.

To facilitate the modeling of complex geometries and examine the interactions among adjacent elements, a two-dimensional Cartesian grid system was developed. The grid was oriented in a longitudinal plane and each element in the grid was uniform in size (7 µm×7 µm). Prior to implantation into the arterial system, the rabbit jugular vein consists of a single layer of SMC, with cells comprising 25% of the volume. As such, that the initial model configuration was comprised of a single layer of elements that were randomly assigned as ECM or SMC in a 3∶1 ratio. With SMC event probabilities dictated by Eq. (4) and (9), the simulation was performed using the following reorganization rules:

- When a new SMC or ECM element is produced, it is assigned to one of the four adjacent spaces. To replicate the wall thickening, reduction in lumen area, and fixed graft length that characterize the process of vein graft remodeling, displaced elements were advanced in the luminal direction (Figure 2A).
- When a SMC undergoes apoptosis or an ECM element is degraded, the resulting void is occupied through movement of the remaining elements in a downward (outside) direction.
- In vivo, shear stress on the luminal surface and intramural wall tension promote continuous movement and reorientation of the cells and matrix elements in the wall, with a configuration that reorganizes to a minimum energy configuration. Regions with a highly focal accumulation of cells or matrix would be sites of elevated stress (tensile or shear), making them unstable and promote reorientation to a low stress state. To simulate the local biologic adaptation that promotes uniform wall thickening, a redistribution algorithm is applied following the loss or gain of an element (Figure 2B). Regions that demonstrate a difference of more than two elements between adjacent columns will be reorganized by the movement of last element in the longer column to the shorter column.

**Figure 3:**
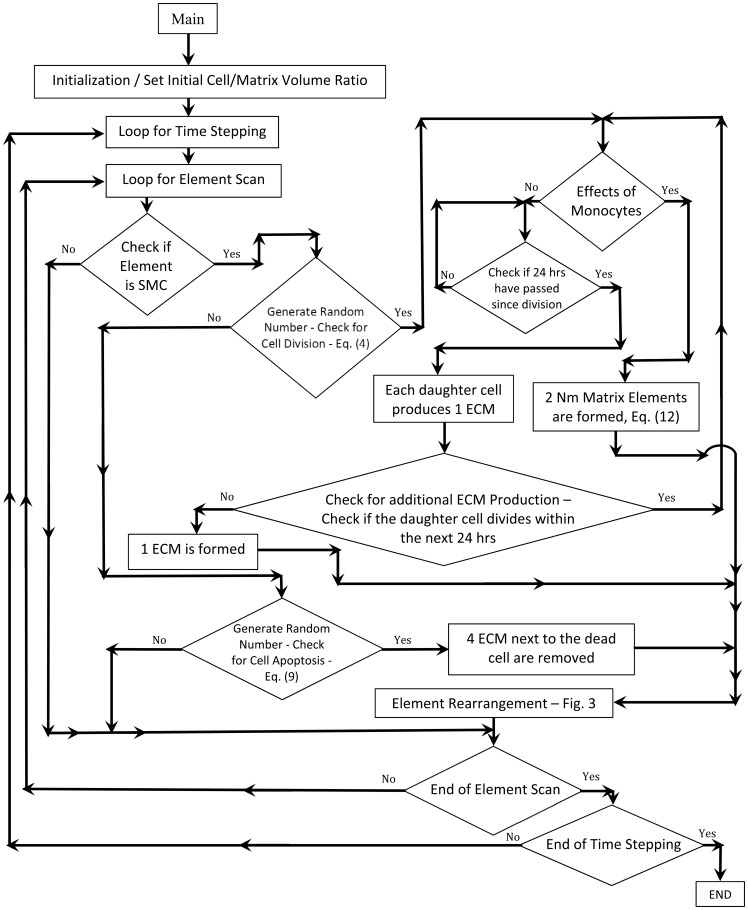
Flow Chart of the Rule-Based Simulation of Vein Graft Remodeling.

Figure 3 shows a detailed flow chart of the agent-based modeling algorithm.

## Results

### Model Validation

**Figure 4:**
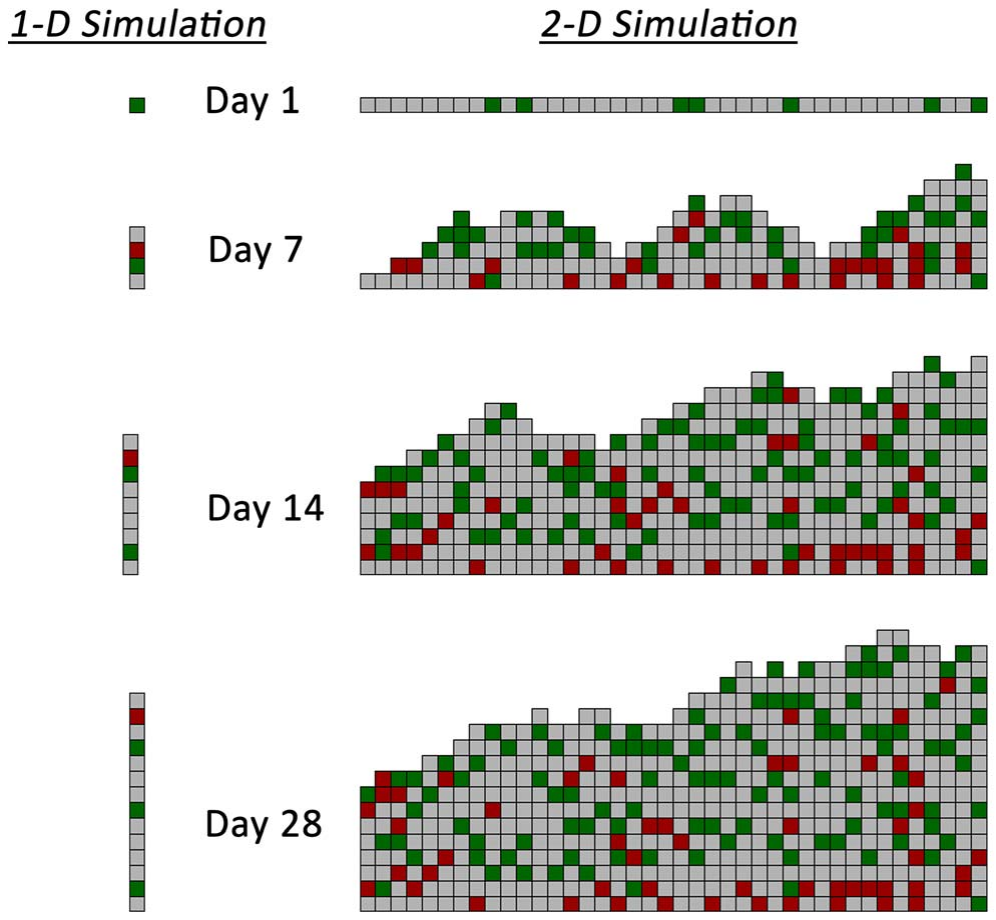
Sample 1-D and 2-D Agent-Based Model Simulation Results. Extracellular matrix (gray), smooth muscle cells (green), and monocytes (red). Wall shear stress is 1.8 dynes/cm^2^ for both simulations.

One- and two-dimensional simulations were performed using a time-step interval of 1 hour and examining initial wall shear stress conditions ranging from 1 to 15 dynes/cm^2^. Sample results at 1, 7, 14 and 28 days following implantation from the one- and two-dimensional models are provided in Figure 4. In both modeling approaches, the accelerated growth phase occurs within the initial two weeks after graft implantation, with modest increases in wall thickness observed in the two- to four-week timeframe. The highest density of monocytes (red cells) is noted in the deepest portions of the intima (i.e. away from the lumen), an observation in agreement with current experimental results [29]. A smooth luminal contour with no more than a single element difference between two adjacent columns is noted and results directly from the redistribution algorithm used in the two-dimensional model (Figure 2B).

**Figure 5:**
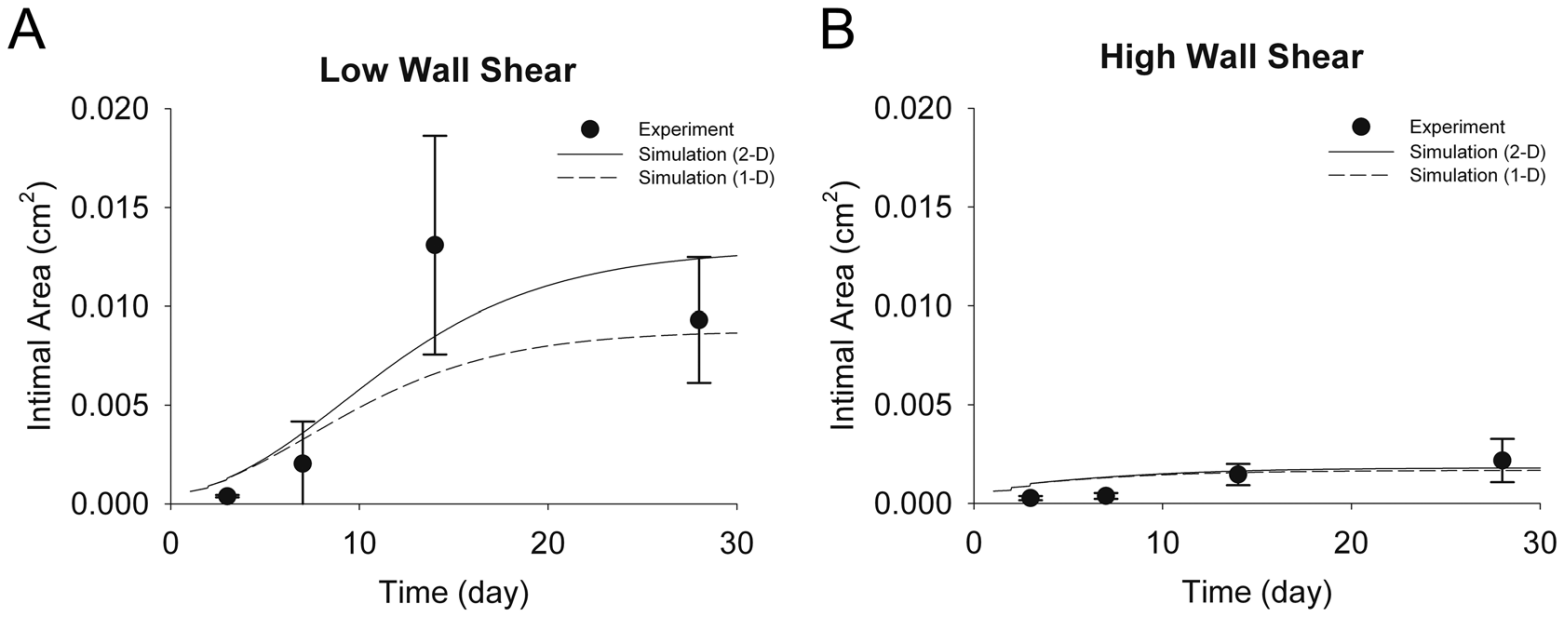
Predicted Intimal Growth for Low and High Flow Conditions. The shear stresses corresponding to low (**A**) and high (**B**) flow conditions are 1.8 and 14 dynes/cm^2^, respectively. The simulation curves are the averages of 10^4^ simulations. Experimental data of intimal area are shown for comparison.

Experimental validation was performed using the distal carotid ligation surgical construct, which creates a unique but reproducible shear environment in each vein graft. Figures 5A and 5B provide the predicted intimal cross-sectional area for the 1-D and 2-D simulations, and the corresponding experimental data, for grafts exposed to low shear (1.8 dynes/cm^2^) and high shear (14 dynes/cm^2^) environments. In order to obtain an equivalent 2-D intimal area, the 1-D intimal area is multiplied by the number of columns (i.e. the number of elements at time zero) in the 2-D domain. The simulation curves shown represent the average of 10^4^ runs.

The results demonstrate that the 1-D simulation can provide an accurate approximation of wall growth at high shear rates, but fails to track the accelerated rates of intimal thickening that are observed in the low shear environment. Under high shear conditions, both the 1-D and 2-D algorithms closely track an early but limited burst in wall growth that abates within 7 days following implantation. Only the 2-D simulation accurately reproduces the sigmoidal shaped curve that characterizes the rapid growth phase that occurs between 7 and 14 days after implantation.

**Figure 6:**
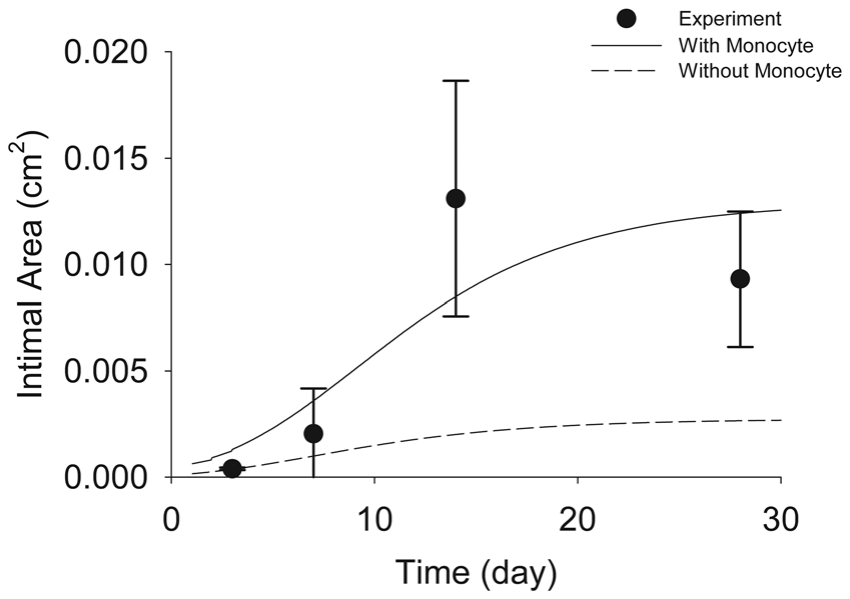
Effect of Monocytes on Intimal Area. The shear stress is equal to 1.8 dynes/cm^2^. Simulation curves are the averages of 10^4^ simulations.

Experimental data across multiple species has convincingly shown the importance of monocyte infiltration in accelerating the hyperplastic response in vein grafts [29], [30]. The performance of the 2-D model was evaluated including and excluding the monocyte module, which acts to augment ECM production. Figure 6 shows the model predicted growth of the intima with and without the monocyte driving term, under low shear (1.8 dynes/cm^2^) conditions. The simulations predict a 79% increase in intimal area at Day 28 when the influence of monocytes is included in the model. Similar to the results described above, inclusion of the monocyte term appears critical to reproducing the sigmoid shaped curve, which most closely tracks the rapid 7 to 14 day intimal growth rate.

### Focal Stenosis Simulation

Following creation of a focal mid-graft stenosis, video microscopy was used to obtain the initial geometry at the time of vein graft implantation. Detailed mapping of the flow field using this initial geometry was obtained using a computational fluid dynamic (CFD) simulation (ADINA R&D Inc., Watertown, MA). CFD simulations used the standardized approaches for incompressible, steady laminar flow with a rigid wall. A plug flow boundary condition was applied to the inlet, with the mean velocity calculated from the flow rate at the time of graft implantation. A two-dimensional axisymmetric geometry was employed to reduce the computational cost, and meshes were generated using curvature size functions to provide increased resolution within the region of the focal stenosis where significant curvature exists. Inlet and outlet extensions (with a length 10 times the graft diameter) were added to facilitate a fully-developed inlet flow condition and to reduce outlet boundary interference, respectively.

**Figure 7:**
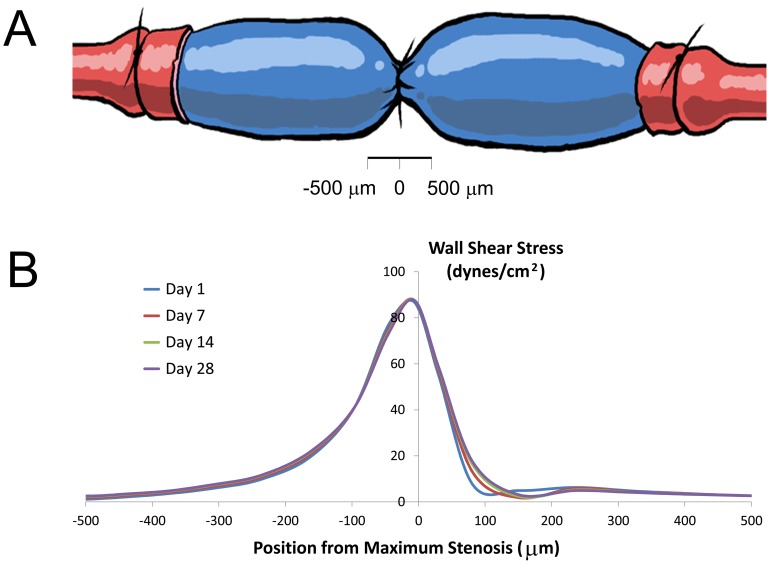
Focal Stenosis Experimental Construct. Schematic of the rabbit focal stenosis vein graft model (**A**) and shear stress distribution along the region of stenosis at 1, 7, 14, and 28 days after implantation (**B**). Computational fluid dynamic methodologies were used to accurately quantify the flow fields within this complex geometry.

**Figure 8:**
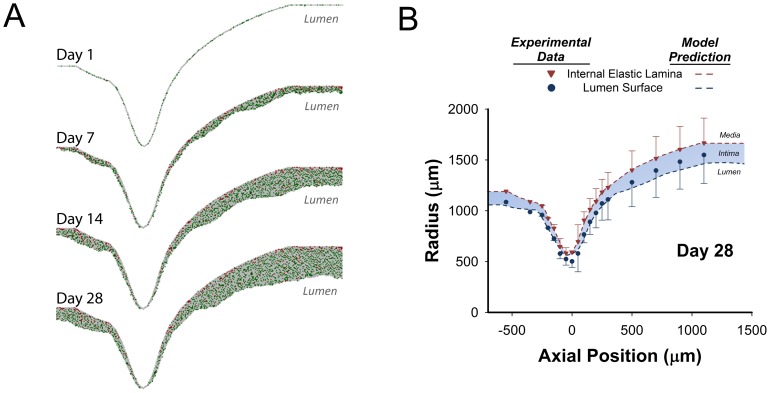
Agent-Based Model Results for a Focal Mid-Graft Stenosis Simulation. **A**. 2-D simulation results at 1, 7, 14, and 28 days after implantation. Extracellular matrix (gray), smooth muscle cells (green), and monocytes (red). **B**. Comparison between simulation results (dashed lines) and experimental data (symbols) for intimal thickening at 28 days following implantation. Model results are the average of 100 simulations and experimental results are an average of 5 independent grafts.

To initiate the agent-based model simulation, the lumen profile (oriented along a longitudinal plane) was placed on a rectangular grid, and the elements through which the curve passed were randomly assigned as either SMC (25%) or ECM (75%). The model simulation was performed using the 2-D algorithm and a one-hour interval time-step. With a modest effect of intimal thickening on wall shear (Figure 7), shear stress profiles within the agent-based modeling algorithm were updated every 7 days. Sample results obtained from the simulation at 1, 7, 14, and 28 days is provided in Figure 8A. Distal to the stenosis, in association with a reduction in wall shear stress, increased intimal thickening is observed at 14 and 28 days after graft implantation.

Following 28 days implantation, lumen surface and IEL geometries obtained from the harvested vein grafts (mean ± SD) and the average results from 100 model simulations (blue shaded region) are provided in Figure 8B. When confronted with the complex surface and flow patterns inherent in the focal stenosis, the rule-based model demonstrates excellent agreement with the experimental results. Notable is the accurate prediction of reduced thickening at the site of maximal stenosis and enhanced hyperplasia distal to the stenosis.

**Figure 9:**
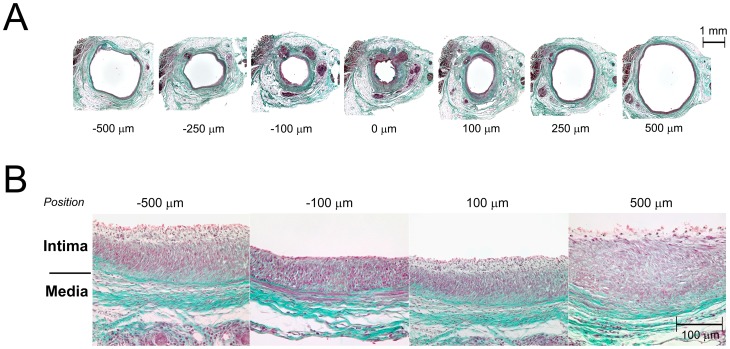
Focal Stenosis Histomorphometry. **A**. Vein grafts harvested at 28 days were serially sectioned along a longitudinal axis and sections collected every 50 µm for morphometric analysis. Representative sections between −500 µm and 500 µm are shown. **B**. High magnification (400x) images of Masson's trichrome stained sections show a highly cellular, poorly organized intima with a sparse network of collagen fibers.

Examination of Masson's trichrome stained specimens prepared from 28-day vein grafts (Figure 9) reveals a high density of disorganized cellular elements within the developing intima. Cells within the intima demonstrate abundant cytoplasm and are interspersed with a loose network of collagen. In contrast, the media is predominately collagen with rare cellular elements that are highly aligned in a circumferential direction.

## Discussion

Rule-based modeling is an appropriate method for simulating multi-scale biological systems in which divergent scale components (such as molecules, cells, tissue, and organs) comprise the system. It enables the observation of the larger scale behavior that is emerging from the interactions among the smaller scale components in the system. The vein graft wall is comprised predominately of smooth muscle cells and extracellular matrix, and the wall remodeling that is observed following implantation results from the complex balance between cell proliferation/apoptosis and matrix synthesis/degradation. Intimal thickening has been simulated in the current study through the application of a set of rules that describe the individual behavior of the smooth muscle cell and matrix elements.

The rules used in development of the agent-based model were almost exclusively derived from experimental data generated in our laboratory. Well-defined experimental constructs and specialized tissue assays were used to maximize the breadth and specificity of the data collected. Despite these efforts, experimental constructs often contain uncertainties and must be carefully analyzed in order to better understand the quantitative values provided by the simulation. In the current study, we utilized a BrdU cell labeling technique to identify cells undergo DNA replication and estimate the probability of smooth muscle cell division. The most uncertain parameter involved in the BrdU analysis scheme is the amount of time BrdU nucleotide is available with the cells for use in DNA synthesis. Phuphanich and Levin [12] measured the half-life of BrdU to be on the order of hours, with some variation depending on the route of administration (intravenous versus oral). In this study, the BrdU availability time and other parameters (cell size and initial volume ratio of cell to matrix) were determined through optimization of the simulation results against the experimentally-derived data.

When the rule-based approach is applied to the simulation of wall thickening around a focal stenosis, it predicts increased intimal growth in regions where the wall shear stress is reduced. Although this is generally consistent with the experimental observations, the model overestimates the actual thickness of the intima. This over-prediction is likely secondary to the uncertainty of the experimental data obtained at Day 14, where the mean intimal area at Day 14 exceeding that observed at Day 28 (Figure 6). The logistic models used for parameter estimation were influenced by this uncertainty and potential overestimation at Day 14, resulting in model simulations that trended towards enhanced intimal growth at Day 28.

As a first generation model that integrates the complexities of both realistic geometries and experimental data, several simplifying assumptions were employed to facilitate construction of the model. Previous work by our group has shown that wall shear is the dominate regulator of intimal growth [16], and experimental and model constructs focused on shear were integral components. In reality, however, changes in vein graft geometry impact the local hemodynamic environment in toto, and wall shear and intramural tensile forces cannot be so cleanly separated. We have recently performed an analysis of the important interactions between wall stress and intramural wall tension (manuscript under review) and have demonstrated notable variations in the ultimate graft morphology based on the dominance of these two driving forces.

The current model is also limited in its focus on intimal growth. Graft remodeling is a combination of both intimal growth and an expansion or contraction of the lumen. As a first generation model, we utilized a fixed Cartesian coordinate grid system where cells and matrix can shift locally but mass movement of these elements does not occur. Integration of lumen remodeling in the agent-based model will require moving to a polar grid system, and inducing *en bloc* movement of the elements based on local forces. Such modifications are currently being incorporated into our second generation model.

Also, this first generation model focuses only on the intima and, outside of monocytes, neglects the contribution to intimal mass induced by movement of external elements into the compartment. SMC migration from the media to intima has been traditionally thought to be the primary source of external cells into the intima. More recently, the movement of myofibroblasts from the adventitia and the transmigration of mesenchymal progenitor cells from the circulation have been identified as important components of the vascular response to injury. All three of these mechanisms are targeted for integration into ongoing model developments.

In summary, the current study is among the first attempts to simulate tissue level vein graft remodeling through the application of basic rules of behaviors to molecular and cellular level activities. Unique to the current study is the careful integration of experimental data at multiple levels of model development and integration. Specifically, we utilize detailed morphometric and histologic analyses from well-characterized vein graft constructs for the direct estimation of model parameters through a wide-range of wall shear stress conditions. The resulting agent-based model is then challenged against an independent set of experiments, where model predictions of intimal growth are evaluated against experimental data obtained from the complex geometry and shear stress patterns offered by a mid-graft focal stenosis.

## Supporting Information

Supplementary Information S1 Derivation of the Probabilities of Cell Division and Apoptosis
(PDF)
