# Supplementary material for: Rule-Based Model of Vein Graft Remodeling
Source: PLoS One. 2013 Mar 22;8(3):e57822. doi: 10.1371/journal.pone.0057822 (PMC3606352; doi:10.1371/journal.pone.0057822)
Supplement: Supplementary Information S1 — Derivation of the Probabilities of Cell Division and Apoptosis (PDF) [file pone.0057822.s001.pdf]

Derivation of the Probabilities of Cell Division and Apoptosis

1. Cell division probability

BrdU gets incorporated into DNA of dividing cells in the S phase of cell cycle. BrdU is injected 24 hours prior to harvest. At harvest, the total number of cells is different from that at the time of BrdU injection because, during the 24 hour period, some cells divided. Thus, at harvest, the % of BrdU stained cells is different from the % of cells (at the time of BrdU injection) which is going to divide during the next 24 hours. We consider the % of cells (at the time of BrdU injection) which is going to divide during the next 24 hours to be cell division probability, and the probability is derived from the observed % of BrdU stained cells at harvest as follows.

First, it is assumed that each of the G1, S, and G2/M phases takes up 1/3 of cell cycle time equally [1]. BrdU availability duration is assumed to be 2 hours [2]. However, for the simplicity of derivation, we consider 8 hours of BrdU availability time first. In Fig. S1, the BrdU availability duration is marked with dark orange color. The four horizontal strips divided into G1, S, and G2/M phases represent the cells undergoing cell division. Even though only 4 strips are shown, cells can be located at any position between the first strip and the 4th strip. The cells located between the 1st and 2nd strips divide during the 24 hour interval because the end of the strip is located in the 24 hour zone. However, those cells do not get stained because S phase is not exposed in the BrdU availability zone. These cell division and staining characteristics are represented by the circles shown in the left and right hand sides of the figure. For those cells located between the 1st and 2nd strips, one open circle before the cell division becomes two open circles after the cell division, which indicates that cells divided during the 24 hour interval, but not stained.

The cells located between the 2nd and 3rd strips divide during the 24 hour interval and get stained because a part or all of S phase is exposed to BrdU availability duration. These cells are represented by one filled circle on the left hand side of the figure (meaning these cells are going to get stained during the 24 hour interval) and two filled circles on the right hand side of the figure (meaning divided and stained). The cells located between the 3rd and 4th strips also divide and get stained during the 24 hour interval. All the other cells are outside of the 24 hour zone, so no division and no staining occur.

Overall, the cells represented by three circles (one open and two filled) on the left hand side of the figure divided, but at harvest, cells represented by 4 filled circles on the right hand side of the figure are observed as stained. The % of cells that were going to divide at BrdU injection can be expressed as a function of the % of BrdU positive cells observed at harvest. From Fig. S1, the total number of cells that were going to divide since BrdU injection is (3/4)*BrdU***Nt* (*BrdU* is the % of *BrdU* stained cells at harvest and *Nt* is the total number of cells counted at harvest), and the total number of cells at BrdU

injection is Nt-(3/4)(BrdU/100)*Nt. Then, the space-averaged probability of cell division can be written as

$$\bar{p}_{division} = \frac{(3/4)(BrdU/100)*Nt}{Nt-(3/4)(BrdU/100)*Nt} = \frac{(3/4)BrdU}{100-(3/4)BrdU} \quad (1)$$

where *Nt* is the total number of cells counted at harvest, and *BrdU* is the % of *BrdU* positive cells observed at harvest. "Space-averaged" means without reference to the location of the cells in the intima.

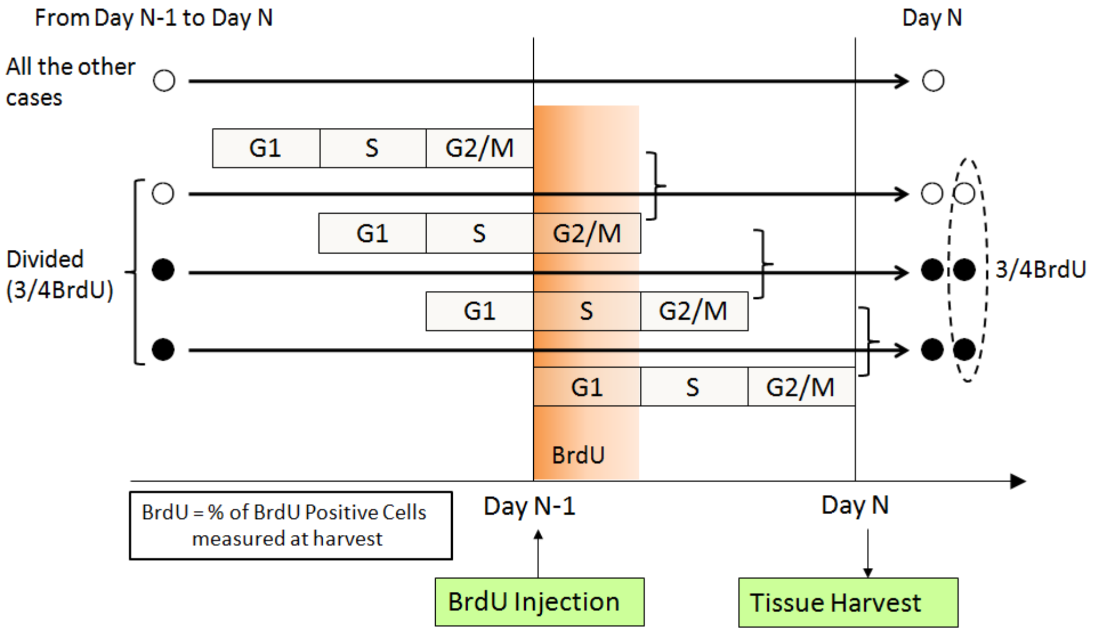

Figure S1. Schematic diagram of cells undergoing cell division between BrdU injection and tissue harvest. Dark orange region represents BrdU availability duration. Four strips divided into three phases (G1, S, and G2/M) represent cells. At the end of each strip, cells divide. Even though only 4 strips are shown, cells can exist at any location between the top and bottom strips. Cells whose S phase is exposed to BrdU availability duration get stained. Open circles on the left hand side of the figure represent the cells that are going to divide during the 24 hour interval, but not going to be stained. Filled circles on the left hand side of the figure represent the cells that are going to divide and get stained during the 24 hour interval. Open circles on the right hand side of the figure represent divided cells but not stained at harvest. Filled circles on the right hand side of the figure represent the divided and stained cells at harvest.

In the case of 2 hours of BrdU availability duration, the number of circles on the right hand side of Fig. S1 between the top and bottom strips of cell cycle is 24 (10 circles are filled ones and 14 circles are open ones). This means the cells corresponding to half of the 24 circles divided while the cells corresponding to the 10 filled circles were observed stained at harvest. Then, the Eq. (1) above becomes

$$\begin{aligned}\bar{p}_{division} &= \frac{(12/10)(BrdU/100)*Nt}{Nt-(12/10)(BrdU/100)*Nt} \\ &= \frac{(1.2)BrdU}{100-(1.2)BrdU}\end{aligned} \tag{2}$$

The space-averaged probability of cell division per 1-hour period is obtained by dividing Eq.(2) by the time between BrdU injection and tissue harvest (T_B):

$$\bar{p}_{division} = \frac{(1.2)BrdU}{100-(1.2)BrdU}\cdot\frac{1}{T_B} \tag{3}$$

For the spatial distribution of cell proliferation, $S_{P_{division}}$, the following form is fitted to experimental data [3].

$$S_{P_{division}}(x) = Ce^{-Dx} \tag{4}$$

where x is the distance from the luminal surface and C and D are constants.

The cell division probability as a function of spatial location is obtained by multiplying Eqs. (3) and (4) adjusting the coefficient C in Eq. (4) such that

$$\begin{aligned}p_{division} &= \bar{p}_{division}\cdot C^{*}e^{-Dx} \\ &= \frac{(1.2)BrdU}{100-(1.2)BrdU}\cdot\frac{C^{*}e^{-Dx}}{T_B}\end{aligned} \tag{5}$$

and

$$\int_0^{IT} p_{division}dx = IT\cdot\bar{p}_{division} \tag{6}$$

where IT is intimal thickness. From Eqs. (5) and (6),

$$\int_0^{IT} C^{*}e^{-Dx}dx = IT \tag{7}$$

$$C^{*} = \frac{IT}{\int_0^{IT} e^{-Dx}dx} = \frac{D\cdot IT}{\left(1-e^{-D\cdot IT}\right)} \tag{8}$$

2. Cell apoptosis probability

It is assumed that the cells undergoing apoptosis remain in the tissue for 24 hours before being removed from the tissue [4]. TUNEL positive cells observed at the tissue harvest are the cells that started apoptosis some time during the last 24 hours. The space-averaged cell apoptosis probability during 1 hour period can be written as

$$\bar{p}_{division} = \frac{TUNEL}{100} \cdot \frac{1}{T_D} \tag{9}$$

where TUNEL is the percentage of TUNEL positive cells at tissue harvest, and T_D is the amount of time the dead cells remain in the tissue before being removed.

For the spatial distribution of cell apoptosis, $S_{P_{apoptosis}}$, the following form is fitted to experimental data [5].

$$S_{P_{apoptosis}}(\hat{x}) = He^{-G\hat{x}} \tag{10}$$

where $\hat{x}$ is the distance from the outside of intima and H and G are constants.

The cell apoptosis probability as a function of spatial location is obtained by multiplying Eqs. (9) and (10) adjusting the coefficient H in Eq. (10) such that

$$\begin{aligned} p_{apoptosis} &= \bar{p}_{apoptosis} \cdot H^{*} e^{-G\hat{x}} \\ &= \frac{TUNEL}{100} \cdot \frac{H^{*} e^{-G\hat{x}}}{T_D} \end{aligned} \tag{11}$$

and

$$\int_0^{IT} p_{apoptosis} d\hat{x} = IT \cdot \bar{p}_{apoptosis} \tag{12}$$

where IT is intimal thickness. From Eqs. (11) and (12),

$$\int_0^{IT} H^{*} e^{-G\hat{x}} d\hat{x} = IT \tag{13}$$

$$H^{*} = \frac{IT}{\int_0^{IT} e^{-G\hat{x}} d\hat{x}} = \frac{G \cdot IT}{\left(1 - e^{-G \cdot IT}\right)} \tag{14}$$

References

1. Yamamoto M, Acevedo-Duncan M, Chalfant CE, Patel NA, Watson JE, Cooper DR (2000) Acute glucose-induced downregulation of PKC-beta II accelerates cultured VSMC proliferation. American Journal of Physiology – Cell Physiology 279: 587-595.

2. Phuphanich S, Levin VA (1985) Bioavailability of bromodeoxyuridine in dogs and toxicity in rats. Cancer Research 45: 2387-2389.
3. Berceli SA, Davies MG, Kenagy RD, Clowes AW (2002) Flow-induced neointimal regression in baboon polytetrafluoroethylene grafts is associated with decreased cell proliferation and increased apoptosis. Journal of Vascular Surgery 36: 1248-1255.
4. Potten C, Wilson J (2004) Apoptosis – the life and death of cells. New York: Cambridge University Press.
5. Furuyama T, Komori K, Shimokawa H, Matsumoto Y, Uwatoku T, Hirano K, Maehara Y (2006) Long-term inhibition of Rho kinase suppresses intimal thickening in autologous vein grafts in rabbits. Journal of Vascular Surgery 43: 1249-1256.
